# Tricks and Tips in Urology: Use of a Semi-rigid Ureteroscope for Super-mini Percutaneous Nephrolithotomy

**DOI:** 10.7759/cureus.117363

**Published:** 2026-10-02

**Authors:** Abdennasser Lakrabti, Ali Akjay, Sohaib Bidan, Jihad El Anzaoui

**Affiliations:** 1 Urology, Moulay Ismail Military Hospital, Meknes, MAR; 2 Urology, Military Hospital Moulay Ismail, Meknes, MAR

**Keywords:** access sheath, mini percutaneous nephrolithotomy, semirigid ureteroscope, stone, technique

## Abstract

Percutaneous nephrolithotomy (PCNL) is a standard technique for the management of renal stones, and its evolution has been marked by progressive instrument miniaturization and advances in laser technology. Super-Mini Percutaneous Nephrolithotomy (SMP) represents a miniaturized form of PCNL characterized by the use of a small-caliber percutaneous access, generally ranging from 10 to 14 Fr. This approach aims to preserve the efficacy of stone fragmentation and removal while reducing renal parenchymal trauma and postoperative morbidity. In this context, the use of a small-caliber semi-rigid ureteroscope through a 10-Fr access sheath represents an attractive alternative when dedicated SMP equipment is unavailable.

The procedure begins with ureteral catheterization using a 7-Fr Chevassu catheter (Coloplast, Humlebæk, Denmark), allowing retrograde pyelography and passive reduction of intrarenal pressure. Percutaneous renal puncture is then performed, followed by progressive tract dilation up to 10 Fr according to the surgeon’s preference.

A 10-Fr access sheath (Innovative access sheath) is subsequently inserted, allowing passage of a 9.5-Fr semi-rigid ureteroscope (Karl Storz, Tuttlingen, Germany). Renal stones, particularly those smaller than 2 cm, are fragmented using a 275-µm Holmium laser fiber (Boston Scientific, Marlborough, USA) in dusting mode.

Intrarenal pressure can be intermittently reduced using aspiration syringes, which also facilitate evacuation of fragments generated during laser lithotripsy.

SMP offers several features that may improve procedural tolerance. The reduced percutaneous tract diameter may decrease renal parenchymal trauma and postoperative blood loss, potentially reducing transfusion requirements and bleeding-related complications. Active aspiration and fragment evacuation are additional features of this technique. Miniaturization may also contribute to shorter hospital stays, reduced analgesic requirements, and faster recovery. In selected patients, a tubeless approach or outpatient management may be possible.

From an economic perspective, SMP assessment should not be limited to the initial cost of specialized equipment. The costs of laser fibers, access sheaths, and miniaturized instruments should be considered alongside potential benefits such as fewer complications, reduced transfusion requirements, shorter hospital stays, and fewer secondary procedures. In resource-limited healthcare systems, reusable instruments and readily available equipment may improve cost-effectiveness. Therefore, using a semi-rigid ureteroscope through a small-caliber access sheath may provide a simple, accessible alternative when dedicated super-mini nephroscopes and specialized access systems are unavailable.

Overall, Super-Mini PCNL appears feasible, effective, and potentially less morbid for managing selected renal stones. Its small percutaneous tract, laser lithotripsy, active fragment evacuation, and potential reduction in postoperative morbidity make it an attractive minimally invasive approach. Moreover, adapting available equipment, particularly a 9-Fr semi-rigid ureteroscope with a 10-Fr access sheath (Innovative access sheath), may facilitate implementation while reducing procedural costs. This approach could represent a practical alternative to dedicated SMP instrumentation, particularly where specialized equipment is limited.

## Introduction

Percutaneous nephrolithotomy (PCNL) has undergone several technical modifications since its initial description, with progressive reduction in tract size aimed at minimizing morbidity while maintaining effective stone clearance. Mini-PCNL is generally performed using a reduced-caliber access sheath, typically ranging from 11 to 20 Fr, with appropriately sized nephroscopic instruments [1,2].

Super-Mini percutaneous nephrolithotomy (SMP) represents a further evolution of miniaturized PCNL, combining a small-caliber percutaneous tract, a dedicated small nephroscope, laser lithotripsy, and an irrigation and aspiration system designed to facilitate active evacuation of stone fragments while maintaining low intrarenal pressure [1,3-8]. Although SMP has gained increasing interest, the availability of dedicated SMP nephroscopes and specialized access sheaths remains limited, particularly in resource-constrained settings.

In this context, we developed a technical modification inspired by the principles of SMP, using a conventional semi-rigid ureteroscope introduced through a small-caliber access sheath. This approach aims to reproduce the practical advantages of a miniaturized tract while relying on equipment that is more commonly available in endourological practice. The present report describes the technique.

## Technical report

The patient was placed in the Valdivia position, following the standard steps of percutaneous nephrolithotomy (PCNL). After induction of general anesthesia, the patient was positioned appropriately, with adequate padding of all pressure points. The operative field was prepared and draped in a standard sterile fashion.

The equipment required for this Super-Mini PCNL technique is illustrated in Figure 1 and includes an 18-G, 20-cm stainless-steel Chiba puncture needle (Coloplast, Humlebæk, Denmark), a hydrophilic guidewire (Boston Scientific, USA), and a 7-Fr Chevassu catheter (Coloplast, Humlebæk, Denmark). Tract dilation was performed using the Amplatz Renal Dilator Set, consisting of 6-10-Fr dilators (Cook Medical, Bloomington, IN, USA). A 10-Fr access sheath (Coloplast, Humlebæk, Denmark, REF: Sheath inner diameter 10(Fr) Sheath outer diameter (12Fr) Length (28cm)), originally designed for flexible ureterorenoscopy, was modified for use in this technique. Before the procedure, renal ultrasonography was used to measure the distance between the skin of the lumbar region and the kidney. This measurement determined the appropriate length of the access sheath segment to be used. In this patient, a 15-cm segment was sufficient. The modified access sheath was subsequently inserted into the renal collecting system (Figure 1). Its internal diameter allowed passage of a 9.5-Fr semi-rigid ureteroscope (Figure 2).

**Figure 1:**
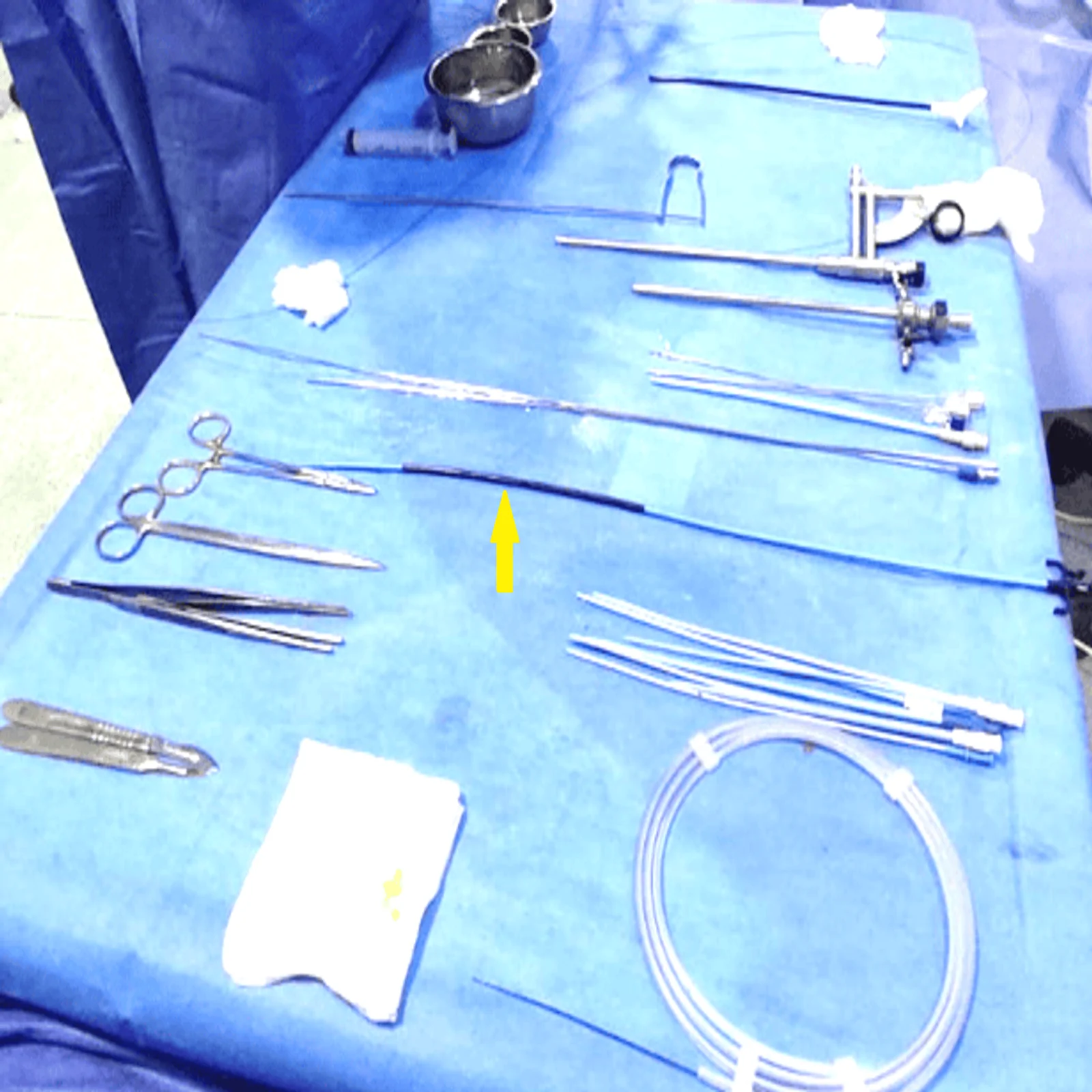
Equipment used for this technique, including the adapted segment of the access sheath (yellow arrow).Equipment used for this technique, including the adapted segment of the access sheath (yellow arrow).

**Figure 2:**
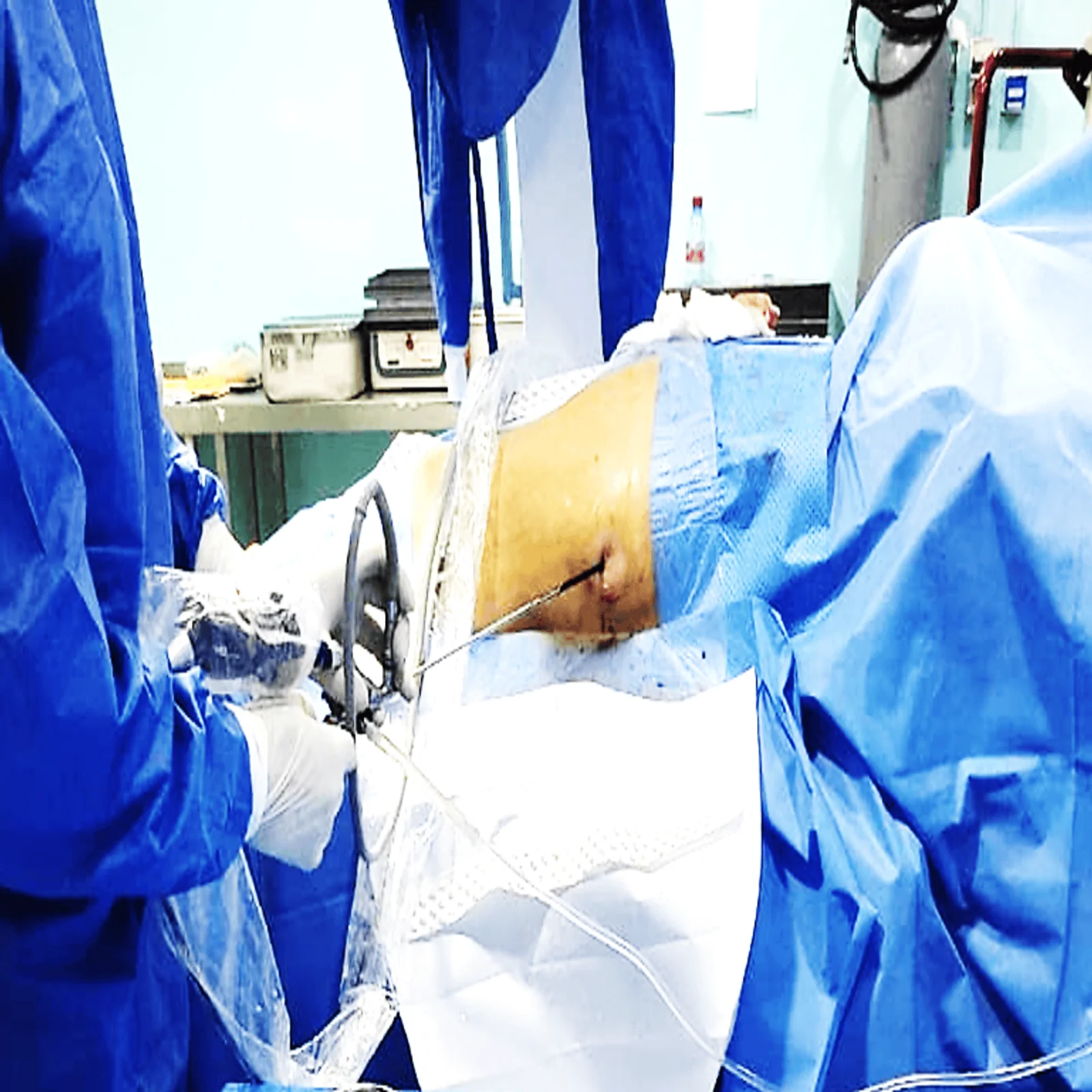
Use of a semi-rigid ureteroscope for our technique (super-mini percutaneous nephrolithotomy).

The semi-rigid ureteroscope used in this technique was manufactured by Karl Storz (Tuttlingen, Germany), reference 27002K. Stone fragmentation was performed using a 275-µm laser fiber (Boston Scientific, Marlborough, USA) connected to a Holmium YAG laser generator (Cook Medical, Germany) in dusting mode.

The procedure began with cystoscopy and insertion of a 7-Fr ureteral catheter into the affected ureter. Retrograde pyelography was performed to delineate the renal collecting system and identify the optimal calyceal target for puncture. The ureteral catheter also allowed passive decompression of the collecting system, thereby reducing intrarenal pressure. During the procedure, intrarenal pressure was further controlled by intermittent aspiration using a syringe through the access system. This aspiration also facilitated evacuation of the stone fragments generated during laser lithotripsy.

Percutaneous renal access was then obtained under fluoroscopic guidance using a standard puncture needle. Once the targeted calyx had been successfully punctured, a hydrophilic guidewire was advanced into the collecting system. The percutaneous tract was progressively dilated using 7-, 8-, 9-, and 10-Fr dilators, after which the 10-Fr access sheath was introduced into the renal collecting system.

A 9.5-Fr semi-rigid ureteroscope was then advanced through the 10-Fr access sheath. This configuration provided direct visualization of the collecting system while maintaining a very small percutaneous tract. Stone fragmentation was performed using a 275-µm laser fiber in dusting mode. During lithotripsy, the “vacuum” effect, resulting from negative-pressure suction, facilitated the evacuation of stone fragments. Intermittent aspiration was performed using a syringe to reduce intrarenal pressure and assist fragment clearance. At the end of the procedure, the collecting system was carefully inspected to identify any residual fragments and to assess the access tract. The decision regarding postoperative drainage was made according to the intraoperative findings and the surgeon’s preference.

Preoperative imaging was performed to assess the renal stone burden, renal anatomy, and collecting system and to facilitate planning of the percutaneous access. Non-contrast computed tomography (NCCT) was used to determine stone size, location, and anatomical characteristics and to identify the most appropriate calyceal target for puncture. Retrograde pyelography was subsequently performed at the beginning of the procedure using the 7-Fr ureteral catheter. This allowed opacification of the collecting system and facilitated fluoroscopic identification of the target calyx. Renal puncture was performed under fluoroscopic guidance according to the principles of standard PCNL access.

Several technical points are important when performing this approach. Correct positioning in the Valdivia position and adherence to the standard sequence of PCNL steps facilitate safe and reproducible renal access. Retrograde pyelography is useful for delineating the collecting system and selecting the appropriate calyx before puncture. Progressive tract dilation should be performed carefully to establish a 10-Fr access tract while maintaining adequate stability of the access sheath. The combination of a 10-Fr access sheath and a 9.5-Fr semi-rigid ureteroscope provides sufficient working space for laser lithotripsy while preserving the advantages of a miniaturized percutaneous tract.

Active aspiration is an important component of this technique. Active intermittent aspiration was performed using a syringe through the retrograde 7-Fr ureteral catheter, rather than directly through the percutaneous access sheath. This aspiration was used to facilitate drainage of the collecting system, assist in fragment evacuation, and contribute to intrarenal pressure control.

The use of a 275-µm laser fiber in dusting mode allows fragmentation of appropriately selected renal stones through the small-caliber access. In the absence of a dedicated Super-Mini PCNL nephroscope and specialized access equipment, the use of a 9.5-Fr semi-rigid ureteroscope through a 10-Fr access sheath represents a practical alternative using readily available instruments. This modification preserves the principles of Super-Mini PCNL while potentially reducing equipment-related costs and maintaining a minimally invasive percutaneous tract.

The patient had a 15-mm lower calyceal stone with a mean stone density of approximately 1,100 HU. The operative time was 60 minutes. Complete stone extraction was achieved, with a 100% stone-free rate confirmed by CT scan at three months postoperatively. No postoperative complications were observed, and the patient was discharged on the second postoperative day.

The procedure was performed in accordance with institutional standards and the principles of the Declaration of Helsinki. Written informed consent was obtained from the patient before the procedure, including consent for the surgical intervention and the use of the described miniaturized technique. Patient confidentiality was maintained throughout the preparation and reporting of the case.

## Discussion

Percutaneous nephrolithotomy (PCNL) has undergone several technical modifications since its initial description. Mini-PCNL consists of performing PCNL using a nephroscope and reduced-caliber access sheaths ranging from 11 to 20 Fr [1,2]. However, over the past decade, two major innovations have revolutionized the surgical management of urinary stones: laser technology and miniaturization (Table 1). The combination of these two approaches has led to the development of flexible ureterorenoscopy, a technique that uses the natural urinary tract, namely the ureter, to introduce a camera into the renal collecting system and fragment stones under direct vision [1,3-8].

**Table 1:** Current Nomenclature of mini-percutaneous nephrolithotomy techniques. PCNL: Percutaneous nephrolithotomy

Technique	Access sheath size (Fr)	Reference
Mini-PCNL	<22	Jackman et al. [1]
Minimally invasive PCNL	9.5–26.0	Nagele et al. [3]
Chinese mini-PCNL	14–20	Li et al. [4]
Ultra-mini PCNL	11–13	Desai et al. [5]
Micro-PCNL	4.8	Desai and Mishra. [6]
Mini-micro PCNL	8	Sabnis et al. [7]
Super-mini PCNL	10–14	Zeng et al. [8]

In this context, micro-percutaneous nephrolithotomy (micro-PCNL) has emerged. This technique retains the principle of creating a percutaneous tract through the skin but introduces a fundamental innovation: minimizing the tract diameter. Stone fragmentation is performed using a laser. Super-Mini PCNL (SMP) is a relatively recent and promising technique that uses a small-caliber access sheath ranging from 10 to 14 Fr [8]. Stone fragmentation is achieved using a laser fiber. One of the specific features of SMP is the combination of passive irrigation with active high-pressure suction, which facilitates evacuation of the fragments generated during laser lithotripsy [9].

The economic impact of miniaturized PCNL should be assessed from a global perspective rather than by considering only the initial cost of the equipment. Although mini-PCNL requires specialized instruments and may involve higher acquisition costs than standard PCNL, its potential economic advantages may result from reduced postoperative morbidity, shorter hospital stays, lower analgesic requirements, and faster recovery.

One of the main advantages of mini-PCNL is the reduction in the diameter of the nephrostomy tract. This may help minimize renal parenchymal trauma and reduce postoperative bleeding. Consequently, the need for blood transfusion and the management of bleeding-related complications may also be reduced. From an economic perspective, avoiding these complications may partially offset the additional costs associated with miniaturized equipment.

Hospital length of stay is another important determinant of the overall cost of PCNL. Mini-PCNL has increasingly been associated with shorter postoperative hospital stays and, in selected patients, with the possibility of a tubeless approach or even outpatient management. Reducing hospital stay may significantly decrease direct treatment costs, particularly in healthcare systems where hospital beds and operating-room resources are limited.

However, the economic evaluation of mini-PCNL should also take into account the costs of single-use devices, laser fibers, access sheaths, and specialized miniaturized nephroscopes. These costs may vary considerably between healthcare systems. In resource-limited settings, the initial investment in miniaturized equipment may represent a major barrier to implementation. Nevertheless, the use of reusable instruments and optimization of operating-room resources may improve the cost-effectiveness of the technique.

Another important consideration is procedural efficacy in terms of the stone-free rate (SFR). An economically advantageous procedure should achieve an acceptable stone-free rate without increasing the need for secondary procedures. If mini-PCNL achieves stone clearance comparable to that of standard PCNL while reducing complications and hospital stay, its cost per successfully treated patient may be lower despite higher procedural costs. Conversely, the need for repeated procedures could offset the economic benefits associated with miniaturization.

In developing countries and resource-limited settings, the economic assessment of mini-PCNL is particularly important. Decisions regarding the adoption of miniaturized techniques should not be based solely on equipment acquisition costs but should also consider hospital stay, complications, blood transfusions, medication requirements, additional procedures, and time to recovery. Thus, a cost-per-successfully-treated-patient analysis may provide a more clinically meaningful assessment than a simple comparison of procedural costs.

To minimize the cost of the surgical procedure, and in the absence of a dedicated Super-Mini PCNL nephroscope and specialized access sheath, our technique may represent a simple and promising alternative. It allows effective stone treatment using readily available instruments while potentially reducing equipment-related costs and maintaining the advantages of a minimally invasive percutaneous approach.

## Conclusions

Super-mini PCNL (SMP) is a safe, feasible, and effective technique for the management of renal stones measuring <3 cm. Its main advantages include a small percutaneous tract, reduced blood loss, high stone-clearance efficacy, and a short operative duration. In the absence of dedicated equipment, the use of a semi-rigid ureteroscope through a small-caliber access sheath may represent a promising alternative, allowing the technique to be performed with readily available equipment while potentially reducing procedural costs and postoperative morbidity.
